# Adult Congenital Permanent Bilateral Dislocation of the Patella with Full Knee Function: Case Report and Literature Review

**DOI:** 10.1155/2012/182795

**Published:** 2012-01-11

**Authors:** Alessandro Bistolfi, Giuseppe Massazza, David Backstein, Stefano Ventura, Raul Cerlon, Maurizio Crova

**Affiliations:** ^1^Department of Orthopaedics and Traumatology, CTO Hospital, Via Zuretti 29, 10126 Turin, Italy; ^2^Mount Sinai Hospital, Department of Surgery, University of Toronto, 600 University Avenue, Suite 476D, Toronto, ON, Canada M5G 1X5

## Abstract

Congenital permanent dislocation of the patella is a rare disorder of the knee joint in which the patella is permanently displaced, even in extension and is fixed on the lateral aspect of the femoral condyle. The dislocation is irreducible without surgical techniques. This rare condition is usually detected within the first decade of life, because of inability of active extension in the knee and impaired ability during walking. This report presents an unusual case of a 51-year-old man with bilateral congenital permanent dislocation of the patella. The pathology had never been treated because there were few symptoms. The patient presented with right knee pain caused by a fall on the knee during his work. The right knee was painful on the lateral side and the clinical signs were positive for pathology of the lateral meniscus, confirmed by MRI. The clinical and the imaging findings suggested a lesion of the lateral meniscus as the probable cause of the pain. Therefore we performed a knee arthroscopy, whose intra-operative findings confirmed the MRI findings. During the surgery we performed just a selective arthroscopic meniscectomy, without correcting patella dislocation, because the condition was unusually asymptomatic before the trauma.

## 1. Introduction

Congenital anomalies of the knee extensor mechanism are rare [1–5]. They can be isolated, associated with other lower limb malformations or part of more complex malformations and dystrophic syndromes (i.e., arthrogryposis, Larsen's syndrome [6], dyschondrosteosis [7], Rubinstein-Taybi syndrome [8], Down syndrome [9, 10], and nail patella syndrome [11]).

Congenital dislocation of the patella (CDP) is a rare condition [5] which must be differentiated from recurrent and habitual patellar dislocations and from some permanent but reducible dislocations due to congenital or genetic causes. In the CDP, a rotational femorotibial displacement is present and it is often associated with varying degrees of proximal tibial epiphyseal metaphyseal lateral torsion. From all the patients with CDP reported in the orthopaedic literature, only a few correspond to the full diagnosis of this condition [1, 12, 13].

This paper presents an unusual case of a 51-year-old man with bilateral congenital permanent dislocation of the patella. The pathology had never been treated because there were few symptoms.

## 2. Case Report

A 51-year-old healthy bricklayer male, born in eastern Europe, presented with right-knee pain caused by a fall on the knee during his work, a few weeks prior. During his childhood, a clinically evident bilateral dislocation of patella was observed and described without the assistance of imaging. Nevertheless, the pathology was asymptomatic, and he never reported difficulty in walking. Therefore, the malformation had never been treated and the patient conducted a normal life including time served in the army.

**Figure 1:**
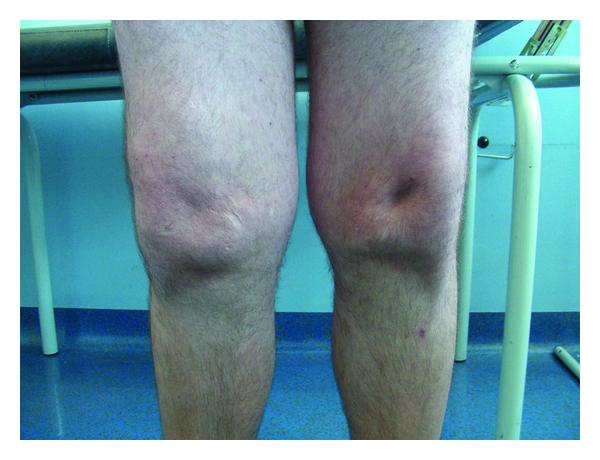
Clinical image of the bilateral dislocated patellae in standing position.

**Figure 2:**
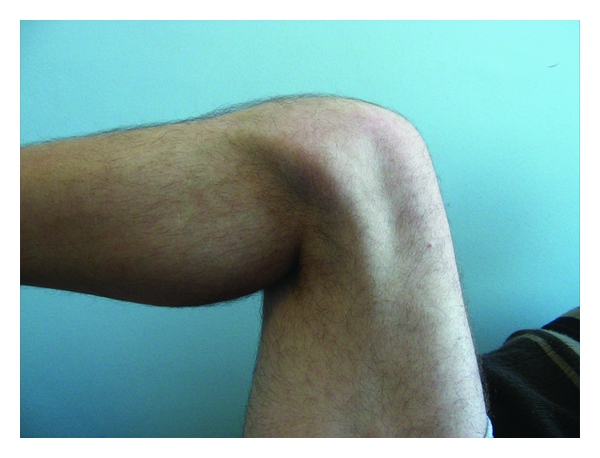
Clinical image of the dislocated patella of the asymptomatic left knee at 90 degrees of flection.

Physical examination revealed that the patient could walk normally, with a very mild stumble on the right knee, which was referred as absent before the trauma. There was conspicuous muscle wasting of each thigh (particularly on the medial side, with a clear depression on the vastus medialis) and 15° of genu valgum. The patellae were lying laterally to the lateral femoral condyle with the knee in the position of full extension (Figure 1) and they displaced more laterally during the flexion (Figure 2). The proximal tibiae were rotated outward. The knees could be extended actively against maximum resistance, with full range of motion. Both the femoral quadriceps worked as knee extensors, and their strength was 5/5. The knees showed no signs of instability or ligamentous deficiency. The right knee was painful on the lateral side, and the clinical signs were positive for pathology of the lateral meniscus.

**Figure 3:**
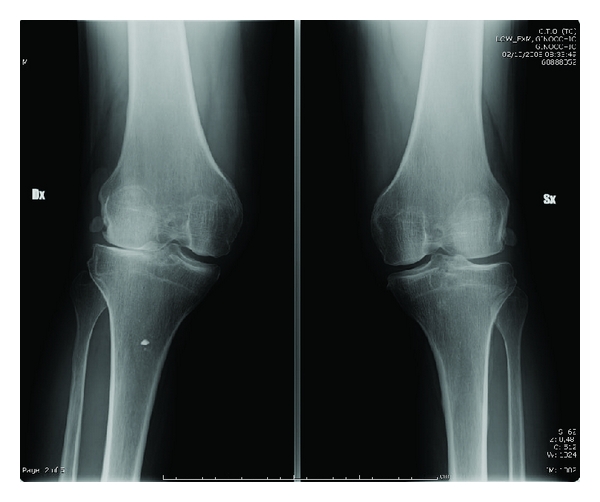
Roentgenograms of bilateral knees in AP, showing laterally displaced patellae with initial degenerative tibiofemoral arthritis.

**Figure 4:**
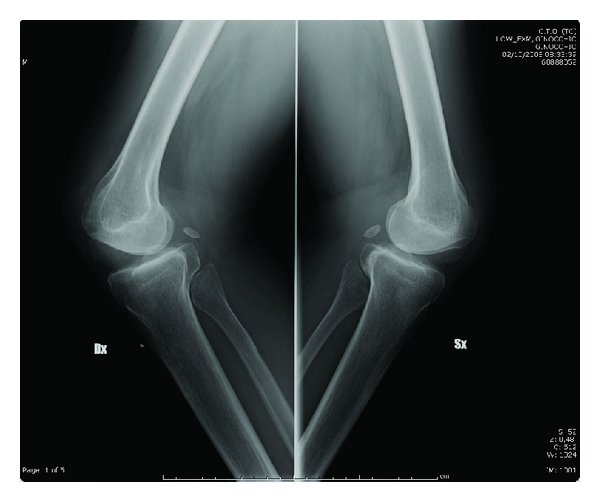
Lateral roentgenograms of the knees at about 30 degrees of flexion, showing dislocated patellae, marked lateral rotation deformities of the tibiae, and condylar deformity.

**Figure 5:**
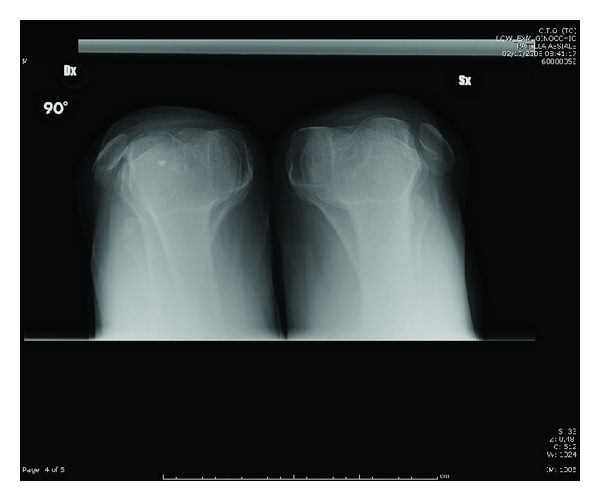
Skyline view roentgenograms, showing an increased deep of the trochlear groove and laterally dislocated patellae.

Roentgenograms of bilateral knees revealed small, laterally displaced patellae with slight degenerative changes in the tibiofemoral joints with subluxated tibia (Figure 3). The medial joint spaces were widened. Lateral roentgenograms showed marked lateral rotation deformities of the tibiae (Figure 4). A skyline view showed an increased deep of the trochlear groove, with laterally dislocated patellae forming a sort of new joint with the lateral aspect of the condyle (Figure 5).

**Figure 6:**
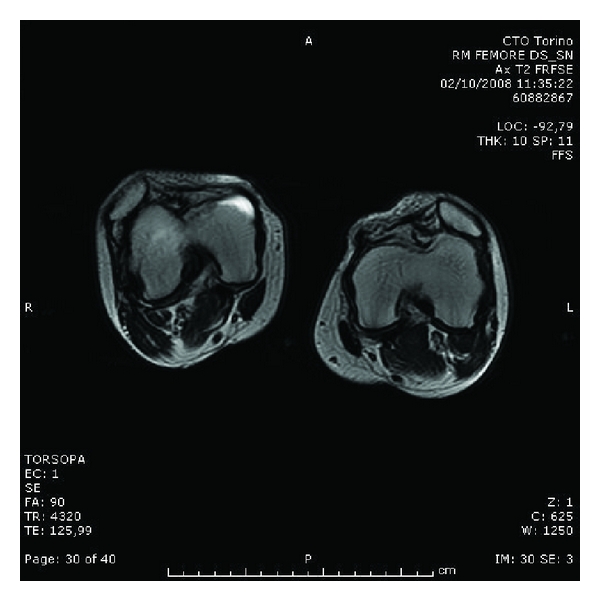
Significative MRI image of the knees (extended), showing the dislocation of the patellae, the severe hypoplasia of the medial side of the femoral quadriceps, and the pseudojoint of the lateral condyles.

Magnetic resonance imaging (MRI) showed a lesion with dislocation of the lateral meniscus on the right knee. On both sides, the MRI confirmed the dislocation of the patellae, a severe hypoplasia of the medial side of the femoral quadriceps, and an external rotation of the proximal tibia particularly evident on the tibial tuberosity (Figure 6).

In order to identify a possible aetiology of the malformation, the patient underwent electromyography/electroneurography, which showed normal function of all the separate portions of the quadriceps muscles thus not explaining the origin of this malformation.

The clinical and the imaging findings suggested a lesion of the lateral meniscus as the probable cause of the pain. Therefore, we performed a knee arthroscopy, whose intraoperative findings confirmed the MRI. During the surgery, we performed just a selective arthroscopic meniscectomy.

After the operation, the patient followed a simple protocol of rehabilitation: 15 days of relative rest with partial bearing and low-molecular-weight heparin therapy, then progressive full recover of activities. Quickly, the knee showed satisfactory improvement, with no pain in walking and a complete range of motion, as before the operation, and comparable to the other knee.

During the last clinical evaluation, 12 months after surgery, written informed consent was obtained from the patient for publication of this case report and accompanying images. A copy of the written consent is available for review by the Editor-in-Chief of this journal.

## 3. Discussion

Congenital permanent dislocation of the patella is a disorder of the knee joint in which the patella is permanently displaced, even in extension, and is fixed on the lateral aspect of the femoral condyle. The dislocation is irreducible without surgical techniques. The disorder is usually bilateral. This rare condition is commonly detected within the first decade of life, because of inability of active extension in the knee and impaired ability during walking [2–4, 14, 15]. The condition may be diagnosed by the flexion deformity of the knee joint [1, 5, 16–21]. Ficat and Hungerford [22] have stated that the presence of fixed-flexion contracture at birth is characteristic of both arthrogryposis and CDP and that, if the former can be excluded, then the latter is likely.

Early diagnosis and definitive correction are generally recommended to prevent subsequent degenerative changes of the knee joint and often to try to restore some knee functionality. Many kinds of surgical treatments have been described [2–5, 15, 23–25].

The case reported here is an unusual and an extremely rare condition in which CDP resulted in no disability. To our knowledge, just a few similar cases have been previously described, but no other cases of completely asymptomatic CDP are reported in literature.

Torisu [26] described a case of a patient with a previously neglected CDP who reported to the surgeon for knee pain caused by a fall with a fracture of the meniscus. The difference is that, in this case, the CDP caused mild disability. Therefore, a correction of the CDP was performed and this surgery gave good results on the functionality. Marmor [27] reported the case of a 63-years-old patient with CDP which gave serious, but not major, disability and was finally treated with a total knee replacement for severe osteoarthritis. Also Bullek et al. [28], Bergquist et al. [29], and Kumagi et al. [30] treated some cases of CDP in adult patients who had osteoarthritis secondary to the deformity with total knee replacement, but, in all these cases, the CDP was treated before the knee replacement with varying results. Robinson et al. [19] reported the case of a 23-years-old patient whose surgical treatment resulted in an acceptable overall functionality, but with a partial inefficiency of the extensor mechanism.

In our case, the patients did not report to the surgeon because of the CDP, but for a very specifical clinical conditions: he described the onset and presented the symptoms of a meniscal rupture, perhaps also suggested by the MRI.

In the end, the arthroscopy confirmed the rupture of the lateral meniscus and the decision to leave the patella in its original, dislocated position ultimately appeared to be prudent and safe.

In our opinion, no significant disability or pain was caused by the CDP before the trauma that caused the lateral meniscus lesion, and, in fact, the recovery after surgery and the good clinical results demonstrate that the CDP, in this patient, was barely asymptomatic.

Nevertheless, during the arthroscopy, a severe degeneration of the articular cartilage was detected. It is therefore possible that the patient could develop early osteoarthritis of the knee, making it similar to the other case as described above. In consideration of the state of the articular cartilage and, most of all, in consideration of the full function of the knees, we decided that surgical corrections of the deformity, such as osteotomies, muscular transposition, and retention, could be ineffective in improving the quality of life and the function of the patient. On the contrary, such a major surgery could destabilise this particular type of knee which developed its own equilibrium during the years. In the future, total knee replacement (TKR) can be performed whenever the patient develops a painful symptomatic osteoarthritis of the knee. Even though this is a rare condition and there are few accounts of total knee replacement for osteoarthritis of the knee associated with CDP, good results of the arthroplasty are reported in each of these cases [27–30].

Usually, in the CDP condition, the strength of the quadriceps acts through an ineffective extensor apparatus and, therefore, during contraction, determines the flexion of the knee instead of the extension. Therefore, the major disability in CDP led back to the extension failure typical of this pathology.

If, for some reason, the ability to extend the knee is preserved, as in the case we observed, CDP does not lead to functional failure, and it is likely that the pathology comes at the attention of the physician during the adulthood of the patient. In this case, the CDP should not be treated until the very probable osteoarthritis, while on the contrary complicate osteotomies and transpositions of muscles and tendons in adult patients usually give poor results on pain and function.
